# Contributions to the faunistics and bionomics of Staphylinidae (Coleoptera) in northeastern  North America: discoveries made through study of the University of Guelph Insect Collection, Ontario, Canada

**DOI:** 10.3897/zookeys.75.767

**Published:** 2011-01-12

**Authors:** Adam J. Brunke, Stephen A. Marshall

**Affiliations:** University of Guelph Insect Collection and Insect Systematics Laboratory, 1216 Edmund C. Bovey Building, School of Environmental Sciences, University of Guelph, Guelph, Ontario, N1G 2W1, Canada.

**Keywords:** rove beetles, exotic species, *Sepedophilus*, *Erichsonius*, *Gabrius*, *Philonthus*, *Quedius*

## Abstract

Staphylinidae (Rove Beetles) from northeastern North America deposited in the University of Guelph Insect Collection (Ontario, Canada) were curated from 2008–2010 by the first author. The identification of this material has resulted in the recognition of thirty-five new provincial or state records, six new Canadian records, one new record for the United States and two new records for eastern Canada. All records are for subfamilies other than Aleocharinae and Pselaphinae, which will be treated in future publications as collaborative projects. Range expansions of ten exotic species to additional provinces and states are reported. The known distributions of each species in northeastern North America are summarized and presented as maps, and those species with a distinctive habitus are illustrated with color photographs. Genitalia and/or secondary sexual characters are illustrated for those species currently only identifiable on the basis of dissected males. The majority of the new records are in groups that have been recently revised, demonstrating the importance of curation and local insect surveys to the understanding of biodiversity, even for taxa and areas considered ‘relatively well-known’.

## Introduction

The rove beetles of northeastern North America, defined here as a region from Ontario eastward and south to Virginia, are well known faunistically compared to other regions of the world, with the exception of the western Palaearctic. For the large subfamilies Staphylininae, Tachyporinae, Steninae, Pselaphinae and to some degree Omaliinae, modern monographs by (Smetana (1971, 1982, 1995), Campbell (e.g. 1973, 1976, 1979, 1982, 1984a, b, 1991), (Puthz (1973, 1974, 1988), (Chandler (1989, 1990), Wagner (1975), and several others have contributed much to our knowledge of their distribution and bionomics. Some diverse groups, including the Paederinae, Oxytelinae, and Aleocharinae, remain poorly known although the North American Aleocharinae are under active study (e.g., Gusarov 2003; Klimaszewski et al. 2006). It is apparent that much remains to be discovered in the northeast, even for those groups that have been recently revised, as evidenced by recent papers on the fauna of the Maritime Provinces of Canada (Klimaszewski et al. 2005, 2007; Majka et al. 2008).

Although the University of Guelph Insect Collection (DEBU) is Canada’s third or fourth largest collection of invertebrates, relatively few of its staphylinid specimens were considered in the course of the above-mentioned revisions. We assume this oversight was due to the collection’s reputation for its coverage of Nearctic and Neotropical Diptera, and the corresponding incorrect assumption that other orders are not well-represented. Although it is true that over half of the 2.5 million or so specimens in the collection are flies, the University of Guelph collection includes several historically important beetle collections and continues to accumulate Coleoptera in the course of ongoing surveys of Ontario’s parks and protected areas as well as routine collecting associated with course work and general collection development. The acquisition of the Alan and Anne Morgan Collection (AAMC) in early 2010 further augmented this material. Previously deposited at the University of Waterloo, this collection of mostly Coleoptera has strengths in the subarctic, boreal and eastern deciduous fauna of Canada. Most Staphylinidae at DEBU were inadequately curated and mostly unidentified prior to recent curatorial work by the senior author, but now all rove beetles in the collection are identified at least to the genus level (except the Aleocharinae and Pselaphinae) and a large proportion of identified specimens are now entered into the central database. We here report on the faunistic discoveries made in the process of curating this material and discuss their importance in the context of previous knowledge. Known distributions are summarized for each species and those species with a distinctive habitus are illustrated to aid in their identification. Where necessary, the aedeagi and/or secondary sexual characters of species are illustrated. Identification of the Pselaphinae and Aleocharinae at DEBU is in progress and future publications on these subfamilies are planned.

## Materials and methods

Specimens were examined with a WILD Heerbrugg M5A stereomicroscope and dissections of male genitalia and genital segments of both sexes were performed in distilled water after preparation following Smetana (1971). All species with a distinct habitus were photographed in dorsal view. All images were prepared using a digital imaging system by Visionary Digital. Maps of the distribution of each species in northeastern North America were prepared using ARC GIS and Adobe Photoshop. Records from the literature at the resolution of state or province are indicated on maps as empty dots, centred on that region. All material examined was from the University of Guelph Insect Collection (DEBU), Ontario, Canada. We follow the taxonomic organization of Newton et al. (2000).

## Results and discussion

### Omaliinae

#### Omalium repandum

Erichson, 1840

##### Materials.

**CANADA: ON: Chatam-Kent Co.**, Tilbury, pitfall trap, 9-VI-1994 (2), T. Sawinksi; **Essex Co.**, Leamington, pitfall trap, 15 to 22-V-1992 (1), 22-V-1992 (1), 9-VI-1993 (1), Palichuck; Point Pelee Natl. Pk., Visitor Centre, malaise and pans, 22 to 29-V-2000, (2), 26-IX to 10-sX-2000 (1) O. Lonsdale. **Huron Co.**, Auburn, Londesboro Rd. nr. Hwy 8, 43.728, -81.529, hedgerow, pitfall, 26-X-2009, A. Brunke (1); Benmiller, Sharpes Creek Line, 43.691, -81.608, hedgerow nr. creek, pitfall, 2-XI-2009, A. Brunke (1); Brucefield, London Rd. nr. Centennial Rd., 43.509, -81.516, hedgrerow nr. creek, pitfall, 27-IX-2009 (1), 12-X-2009 (4) A. Brunke; **Waterloo Reg.**, Blair, Dickie Settlement Rd. nr. WhistleBear golf course, 43.373, -80.400, hedgerow, pitfall, 28-IX-2009 (6), 13-X-2009 (2) A. Brunke; Blair, Dickie Settlement Rd. nr. WhistleBear golf course, 43.373, -80.400, hedgerow, canopy trap in buckthorn, 10-XI-2009, A. Brunke (1); Blair, Fountain St. S. nr Speed River, 43.391, -80.373, hedgerow, pitfall, 24-XI-2009, A. Brunke (1); Blair, Whistlebare Rd. and Township Rd.1, 43.372, -80.362, hedgerow, canopy trap in buckthorn, 18-V-2010, A. Brunke (2); Blair, Whistlebare Rd. and Township Rd.1, 43.372, -80.362, hedgerow, pitfall, 1-VI-2010, A. Brunke (1); Blair, Whistlebare Rd. and Township Rd. 1, 43.367, -80.358, hedgerow, canopy trap in buckthorn, 18-V-2010 (1), 21-IX-2010 (1), A. Brunke; Blair, Whistlebare Rd. and Township Rd. 1, 43.367, -80.358, hedgerow, pitfall, 5-X-2010, A. Brunke (1); St. Jacobs, ‘Stuart pitfall’, 3-VI-1993 (1), 14-VI-1994 (1), T. Sawinksi.

##### Diagnosis.

**Figures 1–6.**
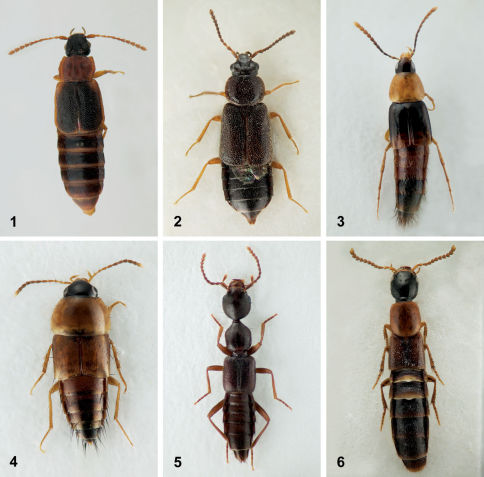
Dorsal habitus. **1** Omalium repandum Erichson **2** Porrhodites fenestralis (Zetterstedt) **3** Ischnosoma flavicolle (LeConte) **4** Tachyporus browni Campbell **5** Eustilicus tristis (Melsheimer) **6** Bisnius cephalicus (Casey).

As the genus Omalium currently lacks a rigorous definition and consists of a heterogeneous assemblage of species (Newton et al. 2000), this species is distinguished from all others of the subfamily occurring in the northeast by the combination of: tarsomere five longer than one to four combined; tarsomeres one to four not conspicuously broadened and without dense setae ventrally; maxillary palpomere three not greatly enlarged relative to segment four (Newton et al. 2000); antennomeres eight to ten elongate; pronotum bright orange with finely crenulate, evenly arcuate margins (Fig. 1).

**Maps 1–4.**
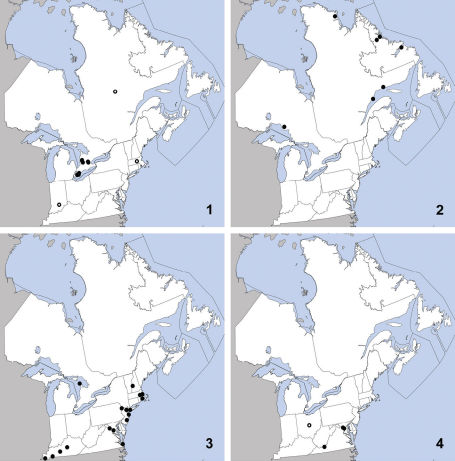
Distribution in northeastern North America, sources of data other than DEBU are quoted in parentheses. **1** Omalium repandum Erichson (Blatchley 1910, Campbell and Davies 1991, Lundgren 1998) **2** Porrhodites fenestralis (Zetterstedt) (Campbell 1984) **3** Ischnosoma flavicolle (LeConte) (Campbell 1991) **4** Sepedophilus campbelli Herman (Campbell 1976, Watrous 2008).

Omalium repandum was previously known from Missouri, South Carolina, Texas (Herman 2001), Indiana (Blatchley 1910), Minnesota, Massachusetts, Georgia (Lundgren 1998) and Quebec (Campbell and Davies 1991). Herein we provide the first specimen-based records of this species in Canada and newly record it from Ontario based on several, relatively recent collections made in southern Ontario (Map 1). The above specimens were caught by passive traps placed in or near forests and thus no microhabitat data was available but Blatchley (1910) reported it as ‘frequent, under dead leaves’. Omalium repandum therefore appears to be forest litter dwelling and is probably widespread in central and eastern North America. The specimens from Huron County, Ontario and the provincial record from Quebec probably represent the northern limit of its range.

#### Porrhodites fenestralis

(Zetterstedt, 1828)

##### Materials.

**CANADA: ON:** **Thunder Bay Distr.** Pukaskwa Natl. Pk. beach trail, dunes, 30-VII-2003, S.M. Paiero (1).

##### Diagnosis.

This species can be readily distinguished from Porrhodites inflatus (Hatch), the only other member of the genus in North America, by the combination of: pronotal margins evenly arcuate; metasternum without microsculpture; antennomere two distinctly longer than segment three (Campbell 1984) (Fig. 2).

Porrhodites fenestralis is a holarctic, boreal to subarctic species known in the Nearctic region from Alberta, British Columbia, Manitoba, Newfoundland, Northwest Territories, Quebec, Yukon Territory, and Alaska, with a relict population in the Rocky Mountains of Montana and Wyoming (Campbell 1984).In the Palaearctic it is known from Austria, Czech Republic, Finland, Germany, Italy, Norway, Russia (North European Territory, Far East, East Siberia, and West Siberia), Sweden, and Switzerland (Smetana in Löbl and Smetana 2004). Ganglbauer (1895) reported it from “Lake Superior”, which could be Michigan, Minnesota or Ontario; the above specimen thus represents the first Ontario record of this species (Map 2). The new locality in Ontario confirms that Porrhodites fenestralis is transboreal in Canada. This species is typically collected in summer to early fall, and most known specimens with collection data were captured in flight; a long series was found swarming on a pine (Pinus)tree (Campbell 1984). Another specimen was found in a deer mouse (Pteromyscus) nest (Campbell 1984).

### Tachyporinae

#### Ischnosoma flavicolle

(LeConte, 1863)

##### Materials.

**CANADA: ON: Bruce Co.**, Dorcas Bay, dunes, pans under malaise, 5 to 13-VI-1999, S.A. Marshall (1).

##### Diagnosis.

Ischnosoma flavicolle is easily distinguished from others of the genus by the distinctly bicolored elytra that each lack a humeral spot (Campbell 1991) (Fig. 3).

This species is primarily southeastern in distribution, with a northward extension along the Atlantic coast, and was known previously from Alabama, Arkansas, District of Columbia, Florida, Georgia, Illinois, Kansas, Kentucky, Louisiana, Maryland, Massachusetts, Mississippi, Missouri, New Hampshire, New Jersey, New York, North Carolina, Oklahoma, South Carolina, Tennessee, Texas, and Virginia (Campbell 1991). Herein we here record this species from Canada (Ontario) for the first time (Map 3). This record represents a significant range expansion and is surprising since the forest of the Bruce Peninsula is known for its dominant boreal elements compared to the more ‘southern’ Carolinian forests in south-western Ontario (Marshall et al. 2001). Throughout its range Ischnosoma flavicolle is frequently collected from sifted litter in a variety of forest types, and hammocks in the southern extremes; it has also been found in grasslands, carrion, under bark (Campbell 1991), and in stream drift (Watrous 2008). The record reported here is from an inland dune on the Lake Huron side of the Bruce Peninsula. Further collecting is necessary to delimit the full range of this apparently widespread species.

#### Sepedophilus campbelli

Herman, 2001

##### Materials.

**UNITED STATES: VA: Giles Co.**, Cascades Recreation Area, sifted from leaf litter in hardwood forest, 11 to 25-V-2008, A. Brunke (1).

##### Diagnosis.

Sepedophilus campbelli is distinguished from other species of the genus in northeastern North America by the combination of: pronotum and elytra without microsculpture and without pale or reddish markings; small size (<1.7mm from the clypeus to the elytral apex); middle-tibia with two apical spines; basal abdominal segments with long lateral bristles.

When Campbell (1976) described this species (under the homonymic name Sepedophilus micans), eight specimens were known from scattered localities in Alabama, Maryland, District of Columbia, and North Carolina. Recently, five specimens of Sepedophilus campbelli were found in Cuivre River State Park, Missouri, at blacklight and under bark (Watrous 2008). Watrous (2008) also noted that Sepedophilus campbelli has been found in Ohio and Florida but without further details. We here report Sepedophilus campbelli as new for Virginia, contributing to the faunistics of this poorly known species (Map 4). Sepedophilus campbelli was recommended for state listing as S3 rank in Missouri based on rarity there and elsewhere (Watrous 2008). Although one specimen has been found on a dead chicken (Campbell 1976), Sepedophilus campbelli is probably a litter or subcortical species.

#### Sepedophilus marshami

(Stephens, 1832)

##### Materials.

**UNITED STATES: NH: Coos Co.**, Jefferson, under bark, 20-IV-2010, T. Murray (1).

**CANADA: ON: Essex Co.**, Kingsville, 14-V-1973, R. Roughly (1); **Waterloo Reg.**, Blair, RARE, Cruickston Creek, yellow pan traps, 15 to 20-VI-2006, S.A. Marshall and M. Bergeron (1); Blair, Dickie Settlement Rd. nr. WhistleBear golf course, 43.373, -80.400, hedgerow, pitfall, 13-X-2009 (1), 10-XI-2009 (1), A. Brunke;Blair, Fountain St. S. nr Speed River, 43.391, -80.373, hedgerow, pitfall, 13-X-2009 (1), 10-XI-2009 (1);Blair, Whistlebare Rd. and Township Rd.1, 43.372, -80.362, hedgerow near soybean field, pitfall trap, 2-XI-2010 (2); **Wellington Co.**, Arkell, 27-IX-1986, L. Work (1); Belwood Lake, lake margin, fallen log overhang, 3-VI-2008, S.A. Marshall (1); Eramosa, Wellington County Rds. 124 and 29, 43.615, -80.215, hedgerow, pitfall, 4-V-2010, A. Brunke (1); Guelph, 19-V-1981, G.M. Grant (1); Guelph, in rotten wood, 20-IV-2007, S.P.L. Luk (1); Guelph, Preservation Park, under bark, 21-IX-2010, S.P.L. Luk (1).

##### Diagnosis.

Sepedophilus marshami may be distinguished from all other members of the genus in eastern North America except Sepedophilus testaceus by the following combination of characters: body size large (>2.3mm from clypeus to elytral apex); tergite seven with a white, apical, palisade fringe and at least one pair of bristles; elytra reddish but without distinct, reddish basal markings; middle-tibia with two apical spines (Campbell 1976). It differs from Sepedophilus testaceus, another exotic species in North America, by the distinctly elongate seventh antennomere, which is subquadrate to weakly transverse in Sepedophilus testaceus. Specimens of Sepedophilus testaceus with rather reddish elytra do exist but these individuals are uniformly pale, while in Sepedophilus marshami the pronotum is distinctly darker than the elytra.

**Maps 5–8.**
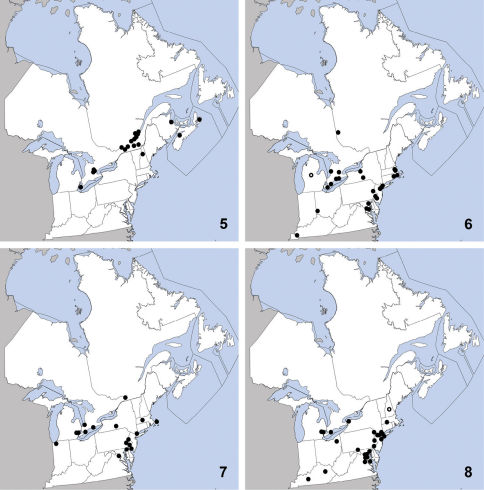
Distribution in northeastern North America, sources of data other than DEBU are quoted in parentheses. **5** Sepedophilus marshami (Stephens) (Campbell 1976, Levesque and Levesque 1995, Majka and Klimaszewski 2008a) **6** Sepedophilus occultus (Casey) (Campbell 1976, Paquin and Dupérré 2001, Sikes 2003) **7** Sepedophilus opicus (Say) (Campbell 1976) **8** Sepedophilus versicolor (Casey) (Campbell 1976).

This exotic, Palaearctic species was first collected in North America in Quebec in 1959 (Campbell 1976) and has since been detected in Nova Scotia (Campbell 1976) and New Brunswick (Majka and Klimaszewski 2008a). It was listed as questionably present in Ontario (Klimaszewski et al. 2010) but herein we confirm its widespread occurrence in the province as early as 1973 (Map 5). We also newly record Sepedophilus marshami for the United States (New Hampshire). This species is widespread in the Palaearctic region (Smetana in Löbl and Smetana 2004). In the Nearctic region, Sepedophilus marshami is typically collected from leaf litter and under loose, fungusy bark in disturbed woodland fragments although it also inhabits debris along freshwater and marine shorelines (Majka et al. 2008) and open areas including raspberry fields and woodland edges (Levesque and Levesque 1995).

#### Sepedophilus occultus

(Casey, 1884)

##### Materials.

**CANADA: ON:** **Huron Co.**, Benmiller, Sharpes Creek Line, 43.691, -81.608, hedgerow nr. creek, canopy trap in buckthorn, 22-VI-2009, A. Brunke (1); **Elgin Co.**, Orwell, 15-VI-1978, D. Morris (1); **Essex Co.**, Point Pelee Natl. Pk., wood area by W beach, malaise/pan traps, 10 to 23-IX-1999, O. Lonsdale (1); **Hald.-Norfolk Reg.**,Turkey Point Provincial Park, malaise trap, 3 to 28-VIII-2009, S. Paiero (1); **Kent Co.**, Rondeau Prov. Park, spicebush trail, 42°18'9N, 81°51'6W, Carolinian forest, WPT, 16to17-VI-2003, Paiero and Carscadden (1); **Wellington Co.**, Guelph, University arboretum nature reserve, ex. Beech litter, 3-V-2009, Brunke and Cheung (1); Guelph, Victoria Rd. and Conservation Line, 43.580, -80.275, hedgerow, pitfall, 2-VI-2009, A. Brunke (1).

##### Diagnosis.

Sepedophilus occultus can be distinguished from other northeastern Sepedophilus with a reddish area at the base of each elytron by the combination of: basal abdominal segments with short bristles only; elytral epipleuron with few or no setae on its basal half; pronotum uniformly colored; elytra without coarse bristles laterally; apical ctenidium of mesotibia restricted to the apex (Campbell 1976). The individual from Kent County has a uniformly reddish body and may be slightly teneral. Similar specimens can be recognized by the unique combination of the elytral epipleuron with few or no setae on its basal half and the impunctate elytral apex.

This species is widely distributed in eastern North America and was previously known from Connecticut, District of Columbia, Georgia, Illinois, Iowa, Kentucky, Maryland, Massachusetts, Michigan, Mississippi, New Jersey, New York, Ohio, Pennsylvania (Campbell 1976), Missouri (Watrous 2008) Rhode Island (Sikes 2003), and Quebec (Paquin and Dupérré 2001). Herein we report it as new for Ontario (Map 6). Sepedophilus occultus is a forest-dwelling species that has been collected mainly from leaf litter and under bark. Its presence in the Boreal Forest Region of Québec (Paquin and Dupérré 2001) is surprising considering its more southern distribution elsewhere but this probably reflects the inadequate knowledge of Canada’s boreal insect fauna rather than a disjunct population.

#### Sepedophilus opicus

(Say, 1834)

##### Materials.

**CANADA: ON: Hald.-Norfolk Reg.**, Backus Tract Woods, sifting leaf litter under mushrooms, 7-VI-2009, A. Brunke and L. DesMarteaux (5); Backus Tract Woods, sifted litter in sugar maple-dominated, mesic forest, 2-IV-2010, A. Brunke (1); **Kent Co.**, Rondeau Prov. Pk., spicebush trail, carolinian forest, malaise, 16 to 29-VII-2003, S. Marshall et al. (1); Rondeau Prov. Pk., south point trail, slough forest, sifting leaf litter, 27-IX-2009, A. Brunke and D.K.B. Cheung (1). **Lambton Co.,** Pinery Prov. Pk., Carolinian Trail, hardwood forest, litter around white pines, 17-IV-2010, A. Brunke (3).

##### Diagnosis.

Sepedophilus opicus can be distinguished from other members of genus in northeastern North America by a combination of: base of elytra with reddish markings extending laterally to the margin; basal abdominal segments with long bristles; elytral epipleuron uniformly setose; pronotal microsculpture distinct (Campbell 1976).

This widely distributed species is known from Alabama, Florida, Illinois, Indiana, Iowa, Maryland, Massachusetts, Michigan, Missouri, New Jersey, New York, North Carolina, Pennsylvania, Québec, Texas, and Virginia (Campbell 1976). Herein we newly record it from Ontario (Map 7). Sepedophilus opicus appears to be confined to deciduous forests in litter, under bark and on mushrooms, and reaches its northern distributional limit in southern Canada. A large number of individuals were found on fresh mushrooms (~35, 5 taken as vouchers) in Backus Tract Woods, Ontario and this may be a preferred microhabitat.

#### Sepedophilus versicolor

(Casey, 1884)

##### Materials.

**CANADA: ON: Kent Co.**, Rondeau Prov. Pk., spicebush trail, Carolinian forest, malaise, 3 to 16-VII-2003 (1), 15-VIII to 7-IX-2003 (1), Marshall et al., 16 to 29-VII-2003, S.A. Marshall (1).

##### Diagnosis. 

Sepedophilus versicolor can be easily separated from others of the genus except Sepedophilus crassus and Sepedophilus ctenidialis by the apical ctenidium of the mesotibia, which extends upwards along the lateral edge (Campbell 1976). It is best distinguished from Sepedophilus crassus and Sepedophilus ctenidialis by the combination of: abdominal sternites four to six with three lateral bristles; abdominal sternites five and six with only one bristle at each side of the midline; smaller size (2.0–2.5mm from clypeus to elytral apex).

This species is broadly distributed in eastern North America and was previously known from District of Columbia, Florida, Georgia, Illinois, Iowa, Kansas, Kentucky, Maryland, Massachusetts, Michigan, Minnesota, Missouri, New Hampshire, New Jersey, New York, North Carolina, Ohio, Pennsylvania, South Carolina, and Virginia (Campbell 1976). Herein we newly record it from Canada (Ontario) (Map 8). This species apparently reaches its northern limit in Ontario’s Carolinian forests. Little is known about its bionomics although it has been found on mushrooms (Campbell 1976) like the related Sepedophilus crassus and in a ‘rotten stump with a small nest’ (Watrous 2008).

#### Tachinus corticinus

(Gravenhorst, 1802)

##### Materials.

**UNITED STATES: MA: Middlesex Co.**, Groton, 22-IV-2010, T. Murray (1).

##### Diagnosis.

Tachinus corticinus is easily distinguished from congeners in northeastern North America by the combination of: pronotum and elytra lacking microsculpture; pronotum with at least borders paler than head; female tergite eight with all lobes of similar size; male sternite seven without apical lobes; small size (3.00–3.75 mm from clypeus to apex of elytra).

**Maps 9–12.**
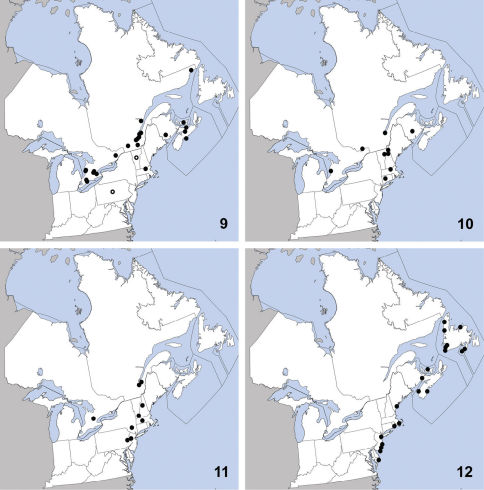
Distribution in northeastern North America, sources of data other than DEBU are quoted in parentheses. **9** Tachinus corticinus (Gravenhorst) (Campbell 1975, Levesque and Levesque 1995, Byers et al. 2000 Majka and Klimaszewski 2008a, Brunke et al. 2009) **10** Tachyporus browni Campbell (Campbell 1979, Klimaszewski et al. 2005) **11** Tachyporus ornatus Campbell (Campbell 1979) **12** Bledius neglectus Casey (Herman 1976, Majka et al. 2008).

This exotic species was first collected in North America in St. Cyrville, Québec in 1967 and was first recognized in North America by Campbell (1975). Since then it has been detected in Vermont, Pennsylvania (Byers et al. 2000), Nova Scotia (Schülke 2006), New Brunswick, Prince Edward Island (Majka and Klimaszewski 2008a) and Ontario (Brunke et al. 2009). Herein we record it as new for Massachusetts (Map 9). Tachinus corticinus is widespread in the Palaearctic region (Smetana in Löbl and Smetana 2004) and has been collected in a variety of open and forested habitats. Although most individuals captured in Hannover, Germany were brachypterous (Assing 1992), Levesque and Levesque (1995) found that nearly all individuals captured in Québec raspberry fields were fully winged. All specimens deposited in DEBU were found to be brachypterous but fully winged individuals do exist in Ontario as Tachinus corticinus was captured in small numbers in raised pan traps (A. Brunke *unpublished data*).

#### Tachyporus browni

Campbell, 1979

##### Materials.

**CANADA: ON:** **Huron Co.**, Benmiller, Sharpes Creek Line, 43.691, -81.608, soybean field, pitfall, 18-IX-2009, A. Brunke (1).

**UNITED STATES: NH: Coos Co.**, Dixville, leaf litter, 6-IV-2010, T. Murray (1); Jefferson, leaf litter, grassy area near stream, 20-IV-2010, T. Murray (1); Dixville, 4-V-2010, T. Murray (4). **MA: Middlesex Co.**, Groton, sifting hay, flood debris in farm field nr. drainage ditch, 30-IV-2010, T. Murray (1). **VT: Orange Co.**, Topsham, sweeping low vegetation, 22-VI-2010, T. Murray (1).

##### Diagnosis.

Tachyporus browni can be easily recognized amongst other northeastern Tachyporus by the combination of a bicolored abdomen and elytra without black discal markings (Fig. 4). Rarely, specimens occur with a small black marking on the scutellum but it does not extend half the length of the elytra as in Tachinus elegans Horn. Additionally, Tachinus elegans lacks dark markings on the pronotum.

This distinctive species was known from 12 specimens at the time of its description, all collected from September to November in southern Québec and Connecticut (Campbell 1979). Klimaszewski et al. (2005) newly recorded it from red spruce-dominated forest in New Brunswick. Herein we newly report Tachyporus browni from Ontario, New Hampshire, Massachusetts, and Vermont (Map 10). Habitat data suggests that Tachyporus browni inhabits moist or wet litter/debris near water. Most of the specimens known are from the cooler months of the year and this seasonality is probably responsible for its rarity in collections. This phenomenon is common for many staphylinid groups (e.g., winter-active Omaliinae in Campbell 1978) and suggests that increased sampling during September to April will yield further discoveries.

#### Tachyporus ornatus

Campbell, 1979

##### Materials.

**CANADA: ON: Wellington Co.**, Belwood Lake, lake margin, fallen log overhang, 3-VI-2008, S.A. Marshall (1).

##### Diagnosis.

Tachyporus ornatus can be distinguished from all other large northeastern members of the genus except Tachinus lecontei, by the combination of a non-bicolored abdomen and the crisp, dark markings on the elytra. From Tachinus lecontei it is most easily identified by the fine microsculpture of the elytra which produces a strong metallic sheen (the former species completely lacks microsculpture).

This species is transcontinental in North America with a disjunct population in the Rocky Mountains of Colorado. It was previously known from the following states and provinces: Alberta, Colorado, Manitoba, Massachusetts, New Hampshire, New Jersey, New York, North Dakota, Pennsylvania, Québec, Saskatchewan, and Vermont. Herein we newly record it from Ontario (Map 11). The only habitat data in Campbell (1979) - “treading under *Alnus*”, and the lakeside habitat of the Ontario specimen recorded here suggest an affinity for decaying organic matter near water, but further collecting is necessary to confirm this.

### Oxytelinae

#### Bledius neglectus

Casey, 1889

##### Materials.

**CANADA: PEI:** Stanhope Beach, National Park, débris vég. sur plage (=beach debris), 23-VII-1979 (2) R. Sexton.

##### Diagnosis.

Bledius neglectus can be identified to the Bledius basalis group of species by the combination of the complete elytral epipleuron, the undivided labrum and the lack of a suture on the epipleuron (Herman 1976). Within this group in northeastern North America, it is best recognized by the combination of: pronotal pubescence directed toward the midline; dark elytral maculation reaching lateral margin of scutellum; basal angle of pronotum strongly sinuate; pronotum with coarse punctures, separated by their widths.

This species is widely distributed along the coast of eastern North America and was previously known from Georgia, Maine, Maryland, Massachusetts, Newfoundland, New Jersey, New York, North Carolina, Nova Scotia, Rhode Island (Herman 1976), and New Brunswick (Majka et al. 2008). Herein we newly report it from Prince Edward Island (Map 12). It occurs along marine coastline on ‘moist, un-vegetated flats’, intertidal zones and often away from shore on the leeward sides of islands and peninsulas (Herman 1976). The specimen from vegetative beach debris in Prince Edward Island may have been a dispersing individual seeking refuge from desiccation.

### Steninae

#### Stenus clavicornis

(Scopoli, 1763)

##### Materials.

**UNITED STATES: MA:** **Middlesex Co.**, Groton, 30-IV-2010, T. Murray, (1).

**CANADA: ON:** **Halton Reg.**, Milton, Derry Rd. and 4_th_ line, grass field, yellow pans, 23 to 24-VI-2001, S. Paiero, (1); **Huron Co.**, Auburn, Hullett-McKillop Rd. nr. Limekiln Line, 43.744, -81.507, soybean field, pitfall, 4-VIII-2010 (1), A. Brunke; Auburn, Limekiln Line, 43.736, -81.506, hedgerow, pitfall, 26-V-2010 (1), A. Brunke; Benmiller, Sharpes Creek Line, 43.691, -81.608, hedgerow nr. creek, canopy trap in buckthorn, 11-V-2009 (2), A. Brunke;Brucefield, London Rd. nr. Centennial Rd., 43.509, -81.516, hedgrerow nr. creek, pitfall, 11-V-2009 (1), A. Brunke; **Ottawa**, Carleton Place, 12-IX-1992, W. Bennett, (1); **Peel Reg.**, Cooksville, pond margin, 30-V-1993, C. Krupke, (1); **Waterloo Reg.**, Blair, Whistlebare Rd. and Township Rd.1, 43.372, -80.362, hedgerow, pitfall, 18-V-2010 (1), A. Brunke; **Wellington Co.**, Guelph, 10-IX-1980, Y. Deedat; Guelph, 17-IX-1980, Y. Deedat; Guelph, University Arboretum, 1-X-2005, M. Bergeron, (1); Guelph, Victoria Rd. and Conservation Line, 43.580, -80.275, soybean field, canopy trap, 23-VI-2009 (1), A. Brunke; **York Reg.**, Stouffville, V-1982, Brian Brown, (1);

##### Diagnosis.

Stenus clavicornis is, at present, only reliably distinguished from congeners in North America by its characteristic aedeagus (Fig. 14).

**Maps 13–16.**
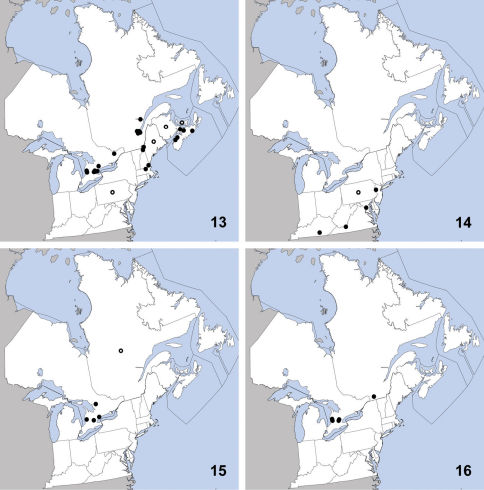
Distribution in northeastern North America, sources of data other than DEBU are quoted in parentheses. **13** Stenus clavicornis (Scopoli) (Puthz 1975, Puthz 1994, Campbell and Davies 1991, Chandler 2001, Majka and Klimaszewski 2008a, Klimaszewski et al. 2010) **14** Eustilicus tristis (Melsheimer) (Melsheimer 1884, Sanderson 1947, Herman 1970) **15** Medon fusculus (Mannerheim) (Campbell and Davies 1991) **16** Scopaeus minutus Erichson (Frisch et al. 2002).

This exotic, Palaearctic species was first recognized in North America by Puthz (1975) based on specimens collected in Québec as early as 1968 in Orsainville. Since then it has been detected in New Brunswick (Campbell and Davies 1991), Prince Edward Island (Klimaszewski et al. 2010), New Hampshire, Pennsylvania (Puthz 1994), Maine (Chandler 2001), and Nova Scotia (Majka and Klimaszewski 2008a). Herein we report Stenus clavicornis from Ontario and Massachusetts on the basis of specimens collected as early as 1980 and 2010, respectively (Map 13). Its native range is very broad and includes most of the Palaearctic region (Smetana in Löbl and Smetana 2004). In North America, this species has been collected from a variety of habitats including open fields, agricultural land, woodlots, and the margins of ponds and salt marshes.

### Paederinae

#### Eustilicus tristis

(Melsheimer, 1844)

##### Materials.

**United States: VA:** **Giles Co.**, Ripplemead, Rte. 460 at bridge, flood debris, 11 to 25-V-2008 (6) A. Brunke.

##### Diagnosis.

Eustilicus tristis is the only northeastern member of the genus and its distinct habitus will readily identify it as a Eustilicus (Fig. 5).

This species is rarely collected and at the time of the most recent revision, it was only known from scattered localities in District of Columbia, Kentucky, New Jersey, Missouri, Oklahoma, Texas (Herman 1970), Arkansas, and South Carolina (Sanderson 1947). Eustilicus tristis was described from ‘Pennsylvania’ by Melsheimer (1844) but no locality was given. Sanderson (1947) stated that it had been recorded from Ohio but we could find no mention of this in the literature. We here newly record it from Virginia (Map 14). This species appears to be a specialist in stream and river drift/flood litter, although it is occasionally found in caves (Peck and Thayer 2003). Watrous (2008) recently recommended Eustilicus tristis for S3 ranking in Missouri based on its specialized, sensitive habitat and general rarity over its known distribution.

#### Medon fusculus

(Mannerheim, 1830)

##### Materials.

**CANADA: ON: Huron Co.**, Auburn, Hullett-McKillop Rd. nr. Limekiln Line, 43.742, -81.514, hedgerow, pitfall, 26-V-2010 (1) A. Brunke; Auburn, Limekiln Line, 43.736, -81.506, hedgerow, canopy trap in buckthorn, 26-V-2010 (2) A. Brunke; Benmiller, Sharpes Creek Line, 43.691, -81.608, hedgerow nr. creek, pitfall, 11-V-2009 (1) A. Brunke; **Muskoka Reg.**, S. Waseosa Rd., 8-VII-1996 (1)W. J. Crins; **Wellington Co.**, Guelph, 26-V-1978 (1) Ron O. Kreazer; Guelph, under rock, 16-III-1983 (1) Brian Brown; Guelph, University Arboretum nature reserve, sifting beech litter, 3-V-2009 (4) A. Brunke and D.K.B. Cheung, sifting litter, 6-VI-2009 (1) A. Brunke; **York Co.**, Toronto, 2-V-1959 (2) R. J. Pilfrey.

##### Diagnosis.

The genus Medon is in need of revision in North America, and Medon fusculus is currently recognizable in North America only from the characteristic modifications of the male seventh sternite and aedeagus (Fig. 15–16).

This exotic, Palaearctic species was first recognized in North America by Campbell and Davies (1991) from Québec but specimen data were not given and the Palearctic species had not yet been revised at that time. Herein we confirm its presence in North America based on comparisons with illustrations in Assing (2004) and newly report it from Ontario based upon specimens collected across southern Ontario as early as 1959 (Map 15). Medon fusculus is widely distributed in the Palaearctic region (Smetana in Löbl and Smetana 2004). In North America, specimens have been sifted from deciduous litter in a small fragment of mature forest and found under a rock. Medon fusculus is a common species in its native range and typically inhabits leaf litter and compost (Assing 2004).

#### Scopaeus minutus

Erichson, 1840

##### Materials.

**CANADA: ON: Huron Co.**, Auburn, Hullett-McKillop Rd. nr. Limekiln Line, 43.742, -81.514, soybean field, pitfall, 23-VI-2010 (6), 7-VII-2010 (6), 21-VII-2010 (4), 1-IX-2010 (1), A. Brunke; Benmiller, Sharpes Creek Line, 43.691, -81.608, hedgerow nr. creek, canopy trap in buckthorn, 22-VI-2009 (1) A. Brunke; Brucefield, London Rd. nr. Centennial Rd., 43.509, -81.516, soybean field, canopy trap, 22-VI-2009 (1) A. Brunke; **Waterloo Reg.**, Blair, Dickie Settlement Rd. nr. WhistleBear golf course, 43.373, -80.400, soybean field, pitfall, 23-VI-2009 (1), 7-VII-2009 (2), 21-VII-2009 (1), A. Brunke; Blair, Fountain St. S. nr Speed River, 43.391, -80.373, soybean field, pitfall, 23-VI-2009 (3),7-VII-2009 (1) A. Brunke; Blair, Whistlebare Rd. and Township Rd.1, 43.372, -80.362, soybean field, pitfall, 13-VII-2010 (4), A. Brunke; Blair, Whistlebare Rd. and Township Rd. 1, 43.367, -80.358, soybean field, pitfall trap, 15-VI-2010 (3), 27-VII-2010 (1), A. Brunke. **Wellington Co.**, Eramosa, Wellington County Rds. 124 and 29, 43.615, -80.215, soybean field, pitfall, 13-VII-2010 (2); Guelph, Victoria Rd. and Conservation Line, 43.580, -80.275, soybean field, pitfall trap, 23-VI-2009 (23), 7-VII-2009 (4), 21-VII-2009 (11), 4-VIII-2009 (1), 18-VIII-2009 (2), 1-IX-2009 (1), 15-IX-2009 (4); Guelph, Victoria Rd. and Conservation Line, 43.580, -80.275, soybean field, vacuum sampled from soybean foliage at 8pm, 16-VII-2009.

##### Diagnosis.

The diverse genus Scopaeus is greatly in need of revision in North America and thus, Scopaeus minutus can only be recognized currently by the form of the aedeagus (Fig. 17).

This exotic Palaearctic species was first reported from North America by Frisch et al. (2002) from Montreal, Québec, Canada; however, no specimen data were provided. Herein we confirm its presence in North America and newly report it from Ontario based on numerous voucher specimens collected from 2009–2010 (Map 16). It is widely distributed in the Palaearctic region (Smetana in Löbl and Smetana 2004). The North American material was collected in passive traps in soybean fields and woodlot edges. Scopaeus minutus is less hygrophilus than others of the genus (Frisch et al. 2002) and is typically found in habitats undergoing early stages of succession (Boháč 1985).

#### Sunius melanocephalus

(Fabricius, 1792)

##### Materials.

**CANADA: ON: Huron Co.**, Auburn, Hullett-McKillop Rd. nr. Limekiln Line, 43.742, -81.514, hedgerow, canopy trap in buckthorn, 26-V-2010 (1); **Waterloo Reg.**, Blair, Whistlebare Rd. and Township Rd. 1, 43.367, -80.358, hedgerow, pitfall, 4-V-2010 (1); **Wellington Co.**, Eramosa, Wellington County Rds. 124 and 29, 43.615, -80.215, hedgerow, pitfall, 4-V-2010 (1).

##### Diagnosis.

Sunius melanocephalus may be easily recognized among other northeastern members of the genus by the combination of the non-serrate lateral margins of the pronotum and the bicolored body.

**Maps 17–20.**
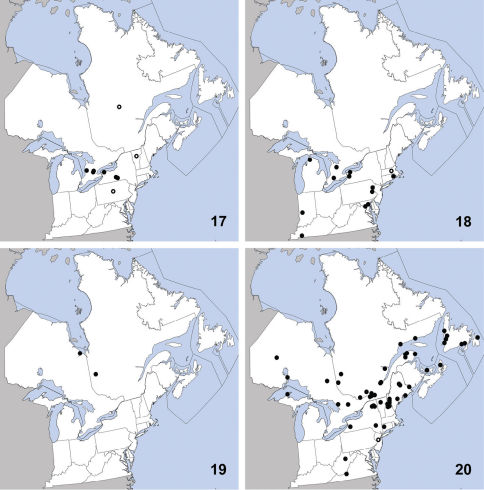
Distribution in northeastern North America, sources of data other than DEBU are quoted in parentheses. **17** Sunius melanocephalus Fabricius (Campbell and Davies 1991, Hoebeke 1991, Byers et al. 2000) **18** Acylophorus agilis Smetana (Smetana 1971, 1978) **19** Bisnius cephalicus (Casey) (Smetana 1995, Paquin and Dupérré 2001) **20** Bisnius siegwaldii (Mannerheim) (Smetana 1995, Paquin and Dupérré 2001).

This species was accidentally introduced from the Palaearctic region to North America and was first recognized on the continent by Hoebeke (1991) from specimens collected in New York as early as 1924. Since then, it has been detected in Pennsylvania, Vermont (Byers et al. 2000), and Québec (Campbell and Davies 1991). As specimen data were not provided for the Québec record, this species’ presence in Canada was uncertain. Herein we verify its occurrence in Canada and newly record it from Ontario based on collections made in 2010 (Map 17); no earlier collections of this species in Ontario were present in DEBU. In the Palaearctic region Sunius melanocephalus is widely distributed (Smetana in Löbl and Smetana 2004) and inhabits a wide variety of habitats including grasslands, swamps, riverbanks, gardens, parks, arable land and mammal burrows (Assing 2008). The Ontario specimens were captured in passive traps at the edges of woodlots.

### Staphylininae

#### Acylophorus agilis

Smetana, 1971

##### Materials.

**CANADA: ON: Middlesex Co.** London, Southern Crop Protection Research Centre, pitfall trap/Masner trap, 21-VI-1995, T. Sawinski (1); **Simcoe Co.**, Noisy River, Prov. Nature Res., beaver lodge, 28-IX-2008, S.A. Marshall (1).

##### Diagnosis.

At present, Acylophorus agilis is reliably separated from others in the diverse Acylophorus pronus - group only by the characteristically shaped paramere of the aedeagus (Fig. 169 in Smetana 1971).

This species is widely distributed in eastern North America, and was previously known from Indiana, Maryland, Massachusetts, Michigan, New York, North Carolina, Pennsylvania, Rhode Island, Missouri, (Smetana 1971), Illinois, and Kentucky (Smetana 1978). Herein we newly record it from Canada (Ontario) (Map 18). Acylophorus agilis has been collected in a variety of periaquatic habitats including ‘floating grass patches’ in a eutrophic pond (Smetana 1971), in sediment-laden debris at the edge of forest creeks (Smetana 1978), at the edge of a sinkhole pond (Watrous 2008), and from debris in a beaver lodge.

#### Bisnius cephalicus

(Casey, 1915)

##### Materials.

**CANADA: ON:** **Cochrane Dist.**, N. Moosonee, sandy beach, ridge along coastal marsh, Picea, Populus, Alnus and herbs, pitfall trap, 23-VI-1990, J. Pilny, (1).

##### Diagnosis.

Bisnius cephalicus is readily distinguished from others of the genus in the northeast by the combination of: body bicolored with orange elytra; pronotum with five punctures in each dorsal row; eyes small, with the space behind them about three times longer (Fig. 6).

At the time of the most recent revision of the genus, this species was known from only two specimens, from Alberta and Manitoba (Smetana 1995). Later, one specimen was collected in the northern Boreal Forest Region of Québec (Paquin and Dupérré 2001). Herein we report the fourth specimen known and newly record Bisnius cephalicus from boreal Ontario (Map 19). This species is apparently transboreal in distribution and its poor representation in collections may be due to a cryptic microhabitat. Its relatively small eyes suggest a subterranean existence in the burrows of mammals, similar to that of certain other Bisnius species.

#### Bisnius siegwaldii

(Mannerheim, 1843)

##### Materials.

**UNITED STATES: VA: Giles Co.**, Mountain Lake, on dead fox squirrel, 11 to 25-V-2008, A. Brunke (3).

**CANADA: PEI:** Long Pond, National Park, milieu marécageux (=marshy environment), 30-VII-1979, R. Sexton (2); West Covehead, débris sur la plage (=beach debris), 25-VII-1979, R. Sexton, (3).

##### Diagnosis.

**Figures 7–12.**
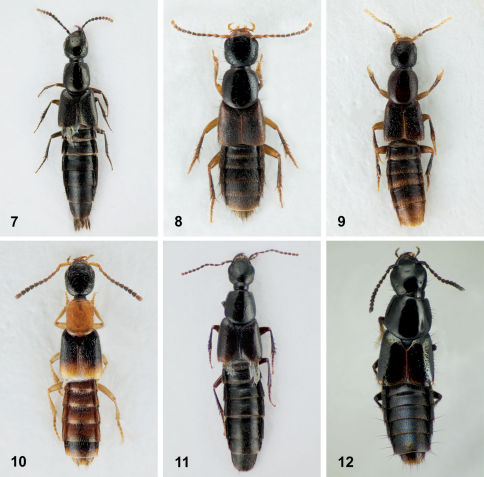
Dorsal habitus. **7** Bisnius siegwaldii (Mannerheim)**8** Erichsonius brachycephalus Frank**9** Erichsonius parcus (Horn) **10** Neobisnius terminalis (LeConte)**11** Philonthus vulgatus Casey**12** Quedius cinctus (Paykull).

Bisnius siegwaldii is easily recognized among other species of the genus in the northeast by the combination of: body not bicolored; elytra dark; pronotum with at least five punctures in each dorsal row; head with punctures arranged to form a ‘V’ (Smetana 1995) (Fig. 7).

This species is transcontinental across northern North America with several collections made further south in both the east and west. It is currently known from Alaska, Alberta, British Columbia, California, Connecticut, Maine, Manitoba, Michigan, Montana, Newfoundland, New Brunswick, New Hampshire, New York, North Carolina, Nova Scotia, Northwest Territories, Ontario, Oregon, Québec, Saskatchewan, Tennessee, Vermont, Washington, West Virginia, Wisconsin, and Yukon Territory (Smetana 1995). Herein we newly record it from Prince Edward Island and Virginia (Map 20). Bisnius siegwaldii is a common species found in carrion, dung, rotting fungi, decaying plant matter and wood, moss, and in vegetation near water (Smetana 1995).

#### Erichsonius brachycephalus

Frank, 1975

##### Materials.

**CANADA: ON: Huron Co.**, Brucefield, London Rd. nr. Centennial Rd., 43.509, -81.516, hedgerow nr. creek, canopy trap in buckthorn, 11-V-2009, A. Brunke (1); **QC:** **La Valleé-de-la-Gatinaeu**,Martindale, hutte à castor (=beaver lodge), 19-IX-1976, R. Sexton (26).

##### Diagnosis.

This species is easily recognized among others of the genus with a sparsely punctate forebody by its large size (>4.7mm from clypeus to abdominal apex) and transverse head with slightly converging temples (Fig. 8).

**Maps 21–24.**
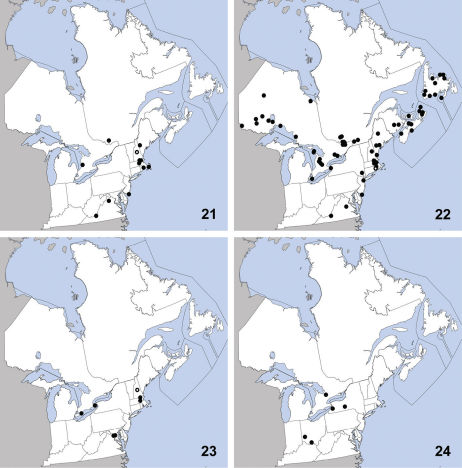
Distribution in northeastern North America, sources of data other than DEBU are quoted in parentheses. **21** Erichsonius brachycephalus Frank (Frank 1975, 1981a, Chandler 2001) **22** Erichsonius nanus (Horn) (Frank 1975, 1981a, Sikes 2003, Klimaszewski et al. 2005) **23** Erichsonius parcus (Horn) (Frank 1975, Chandler 2001) **24** Gabrius amulius Smetana (Smetana 1995).

Erichsonius brachycephalus was previously known from Illinois, Maine, Massachusetts, New Jersey, Texas (Frank 1975),Virginia (Frank 1981a), and New Hampshire (Chandler 2001). Watrous (2008) reported it from Missouri based on specimens collected in stream drift and leaf litter. Herein we newly record it from Canada (Ontario and Québec) and suggest that this species is broadly distributed in northeastern North America, reaching its northern limit in southernmost Canada (Map 21). All available habitat data suggest that this species is strongly associated with decaying vegetative debris along the edges of ponds, lakes, streams, and rivers. Despite  the long series from Quebec beaver lodges, we have not found this species during our searches of beaver lodges in Ontario.

#### Erichsonius nanus

(Horn, 1884)

##### Materials.

**CANADA: PEI:** Long Pond National Park, milieu marécageux (=marshy environment), 30-VII-1979, R. Sexton (2).

##### Diagnosis.

Erichsonius nanus can be recognized among the other densely punctate species in northeastern North America by its larger size (>4.4mm, from clypeus to abdominal apex), and the apical portion of the median lobe of the aedeagus, which is distinctly thin and sinuate in lateral view (Fig. 18).

This species is widely distributed and was previously known from Alaska, British Columbia, Illinois, Maine, Massachusetts, New Brunswick, New Hampshire, Newfoundland, New Jersey, New York, Northwest Territories, Nova Scotia, Ontario, Québec, Washington, Wisconsin (Frank 1975),Virginia (Frank 1981a), and Rhode Island (Sikes 2003). Herein we newly report this species from Prince Edward Island (Map 22). Erichsonius nanus is found in a variety of habitats near water and can be collected in great numbers by sifting or treading the debris present there.

#### Erichsonius parcus

(Horn, 1884)

##### Materials.

**CANADA: ON: Kent Co.**, Rondeau Prov. Pk., spicebush trail, Carolinian forest, yellow pans, 25-V-2003, M. Buck and S. Paiero (1); Rondeau Prov. Pk., spicebush trail, Carolinian forest, yellow pans, 16 to 17-VI-2003, Buck and Carscadden (1); Wainfleet bog, 8km S of Welland, DIE pt. 1 –north ditch (control), 10 to 24-V-1988, A. Stirling (1); 7 to 13-VI-1988, all collected by A. Stirling: D2H pt. 2 – ‘1962 zone’(1), D4H pt. 4 – ‘1980 zone’ (1), D5H pt. 5 – ‘1985 zone’(1), D5E pt. 5 – ‘1985 zone’ (1).

##### Diagnosis.

Erichsonius parcus can be easily distinguished from other northeastern species of the genus with a sparsely punctate forebody, with the exception of Erichsonius pusio, by its small size (<3.6mm from clypeus to abdominal apex). Frank (1975) stated that Erichsonius parcus could be separated from Erichsonius pusio by the paler coloration, the smaller eyes and the head broader behind the eyes. Head shape was found to be highly variable in both males and females of Erichsonius parcus and this character should not be used to identify this species. However, the length of the pronotum appears to be a reliable character and is subequal to that of the elytra in Erichsonius parcus (Fig. 9) and clearly shorter in Erichsonius pusio. It should also be noted that the parameres of the aedeagus in Erichsonius parcus possess characteristic short, stout setae (Fig. 19) which were not illustrated by Frank (1975) but mentioned later in Frank (1981a). Erichsonius pusio lacks these setae on the parameres.

This species was previously known from Florida (Frank 1981), District of Columbia, Massachusetts, Louisiana, Virginia (Frank 1975), and New Hampshire (Chandler 2001). Herein we record it as new from Canada (Ontario) based on eight specimens, all collected in the Carolinian region of southern Ontario (Map 23). Erichsonius parcus has previously been collected by ‘sifting’, in ‘drift’ (Frank 1975) and at lights (Frank 1981a); all Canadian specimens were collected in wet habitats with abundant moss (bog, slough forest). In a survey of southern Ontario peatlands, Blades and Marshall (1994) reported Erichsonius pusio from Wainfleet bog based on a series of specimens found by the first author to be misidentified Erichsonius parcus. To our knowledge, no true Erichsonius pusio have been found in southern Ontario peatlands.

#### Gabrius amulius

Smetana, 1995

##### Materials.

**CANADA: ON: Simcoe Co.** Midhurst, forest nr. Neretva St., under bark of large beech trunk, 4-IX-2009, A. Brunke and K. Brunke (1).

##### Diagnosis.

Gabrius amulius may be recognized by the combination of: large size (at least 5.0mm long from clypeus to abdominal apex); eyes large, with temple that is distinctly less than twice as long as the eye; forebody without a greenish metallic lustre; elytra with sparsely distributed punctures that are separated by two to three times their diameter; area between basal lines on tergites two and three punctate

This apparently rare species was known from only five specimens at the time of its description (Smetana 1995) from localities in New York and Ohio. It was collected in Missouri by Watrous (2008) in a flight intercept trap. Herein we newly report it from Canada (Ontario) (Map 24). All known specimens with microhabitat data were collected in deciduous forests in litter, or in the proximity of decaying wood. Gabrius amulius is almost certainly an uncommon specialist of deciduous or mixed forests.

#### Gabrius appendiculatus

(Sharp, 1910)

##### Materials.

**CANADA: ON: Huron Co.**, Auburn, Hullett-McKillop Rd. nr. Limekiln Line, 43.744, -81.507, soybean field, pitfall, 23-VI-2010 (1), 7-VII-2010 (2), A. Brunke; Auburn, Limekiln Line, 43.736, -81.506, soybean field, pitfall, 23-VI-2010 (1), 7-VII-2010 (1), 4-VIII-2010 (1), 18-VIII-2010 (1), A. Brunke; **Waterloo Reg.**, Blair, Dickie Settlement Rd. nr. WhistleBear golf course, 43.373, -80.400, soybean field, pitfall, 23-VI-2009 (1), 7-VII-2009 (1), 21-VII-2009 (6), 4-VIII-2009 (1), A. Brunke; Blair, Dickie Settlement Rd. nr. WhistleBear golf course, 43.373, -80.400, hedgerow, canopy trap in buckthorn, 27-X-2009, A. Brunke (1); Blair, Fountain St. S. nr Speed River, 43.391 -80.373, soybean field, canopy trap, 15-IX-2009, A. Brunke (1);Blair, Whistlebare Rd. and Township Rd.1, 43.372, -80.362, hedgerow, pitfall, 4-V-2010, A. Brunke (1); Blair, Whistlebare Rd. and Township Rd.1, 43.372, -80.362, soybean field, pitfall, 29-VI-2010, A. Brunke (1); Blair, Whistlebare Rd. and Township Rd. 1, 43.367, -80.358, soybean field, pitfall trap, 13-VII-2010 (1), 25-VIII-2010 (1), A. Brunke; **Wellington Co.**, Eramosa, Wellington County Rds. 124 and 29, 43.615, -80.215, hedgerow, pitfall, 4-V-2010, A. Brunke (2); Eramosa, Wellington County Rds. 124 and 29, 43.615, -80.215, soybean, pitfall, 15-VI-2010 (3), 29-VI-2010 (3), A. Brunke; Guelph, Victoria Rd. and Conservation Line, 43.580, -80.275, soybean field, pitfall, 21-VII-2009 (1), 4-VIII-2009 (6), 1-IX-2009 (4), A. Brunke; Guelph, Victoria Rd. and Conservation Line, 43.580, -80.275, soybean field, canopy trap, 4-VIII-2009 (1), 18-VIII-2009 (1), A. Brunke

##### Diagnosis.

Gabrius appendiculatus can be distinguished from congenersin the northeast by the following combination of characters: area between basal lines on tergites two and three impunctate; basal antennomeres not distinctly paler than rest of antenna; males with sternite eight broadly notched; females with tergite 10 pointed (as opposed to truncate) apically.

**Maps 25–28.**
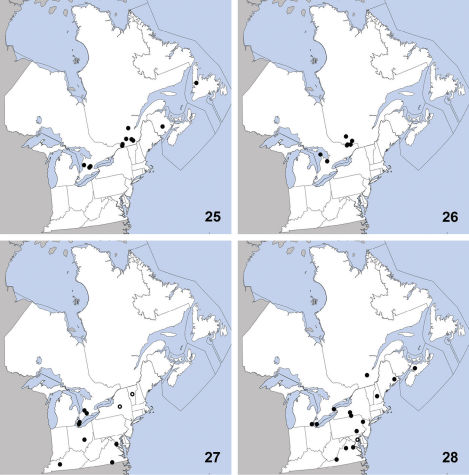
Distribution in northeastern North America, sources of data other than DEBU are quoted in parentheses. **25** Gabrius appendiculatus (Sharp) (Smetana 1995, Klimazewski et al. 2005) **26** Gabrius vindex Smetana (Smetana 1995) **27** Neobisnius occidentoides Frank (Frank 1981b) **28** Neobisnius terminalis (LeConte) (Frank 1981b).

This Palaearctic species was first recognized in North America by Smetana (1989) (as Gabrius subnigritulus (Reitter)) based on specimens collected as early as 1978 in Ormstown, Québec and from other localities in British Columbia and Newfoundland; the earliest records from the west coast of North America were from 1979. Klimaszewski et al. (2005) newly recorded it from red spruce-dominated forests in New Brunswick. Herein we report the dispersal of Gabrius appendiculatus into the southern portion of Ontario from specimens collected in 2009–2010 (Map 25). No other specimens before this period are known to have been collected in Ontario. In its native range, Gabrius appendiculatus is widespread in distribution (Smetana in Löbl and Smetana 2004). Numerous specimens collected from soybean fields in Ontario further support the conclusion of Smetana (1989) that this species is well-adapted to agricultural habitat in North America.

#### Gabrius vindex

Smetana, 1995

##### Materials.

**CANADA: ON: Bruce Co.**,Bruce Pen. Natl. Pk., Upper Andrew Lake, beaver lodge, 26-VII-2008, S. A. Marshall (1); **Simcoe Co.**, Noisy River Prov. Pk., Res., beaver lodge, 28-IX-2008, S.A. Marshall (1).

##### Diagnosis.

Gabrius vindex is easily distinguished from all other species of the genus in northeastern North America except Gabrius astutoides by the combination of: punctures of the elytra dense, separated by their widths or less; size large (at least 6.0mm from clypeus to abdominal apex); the area between the basal lines of tergites two and three punctate. From Gabrius astutoides it is most easily separated by the shape of the pronotum which is narrowed anteriorly in Gabrius vindex and parallel in Gabrius astutoides.

This species is transcontinental in northern North America and was previously known from Alaska, Manitoba, Minnesota, and Québec (Smetana 1995). Herein we newly record Gabrius vindex from Ontario (Map 26), representing the only known localities in northeastern North America other than the Gatineau area of Québec. All known specimens of Gabrius vindex have been collected in debris adjacent to the water’s edge, and Smetana (1995) suggested that beaver lodges provide ideal conditions for this species. The Ontario specimens and the 92 DEBU specimens from beaver lodges in the Gatineau area of Québec strongly support this.

#### Neobisnius occidentoides

Frank, 1981

##### Materials.

**CANADA: ON: Essex Co.**, East Sister Is. Prov. Nature Res., dry pond bed, yellow pans, 30-VII-2003, S.A. Marshall (1); Leamington, pitfall trap, 17-VIII-1993 (1); **Huron Co.**, Centralia, Dev 1A, pitfall, 16-VIII-1992 (1); **Middlesex Co.**, London, southern crop protection research centre, corn pitfalls 3, 19-VII-1993 (1); London, southern crop protection research centre, pitfall/Masner trap, 2-VIII-1995, T. Sawinski (1).

##### Diagnosis.

This species can be distinguished from other orange and black Neobisnius in northeastern North America by the combination of head completely lacking microsculpture, elytra with apically paler area limited to a narrow strip, and the maxillary palpi with at least one segment darkened.

Neobisnius occidentoides is a widespread species and was previously known from Alberta, Alabama, Arkansas, Arizona, California, Colorado, Idaho, Illinois, Kansas, Kentucky, Louisiana, Manitoba, Minnesota, Mississippi, Missouri, Montana, Nebraska, Nevada, New Mexico, New York, North Carolina, North Dakota, Ohio, Oklahoma, South Dakota, Tennessee, Texas, Utah, Vermont, Virginia, Washington, and Wyoming (Frank 1981b). This species is also known from Mexico. Herein we newly record it from eastern Canada based on several collections made in the Carolinian region of southern Ontario (Map 27). Neobisnius occidentoides is less strongly associated with water margins than are other bicolored species of the genus, and is frequently collected in agricultural fields with moist soil (Frank 1981b).

#### Neobisnius terminalis

(LeConte, 1863)

##### Materials.

**CANADA: ON: Essex Co.**,Middle Is., shore, yellow pans, 11-VI-2003, S.A. Marshall (1); **Niagara Reg.**, Grimsby, J. Pettit (1).

##### Diagnosis.

Neobisnius terminalis is easily recognized among other orange and black species of the genus in northeastern North America by the elytra with a broad, pale apical area (Fig. 10). In other northeastern species, this pale area is restricted to a narrow strip.

This species was previously known from Arizona, California, Colorado, Iowa, Maine, Maryland, Michigan, New Hampshire, New Mexico, New York, Nova Scotia, Pennsylvania, Québec, Texas, and Virginia (Frank 1981b). Herein we newly record it from Ontario (Map 28). It is also known from Costa Rica, Guatemala, Mexico, and Panama (unverified record) (Frank 1981b). Unlike Neobisnius occidentoides, Neobisnius terminalis is strongly associated with the margins of rivers and lakes and is found in litter or under debris. The specimen recorded here from Ontario’s Middle Island (in Lake Erie) was taken in pan traps on a gravel lake shore.

#### Philonthus couleensis

Hatch, 1957

##### Materials.

**CANADA: PEI:** Brackley Beach, National Park, milieu marécageux (=marshy environment), 2-VIII-1979, R. Sexton (1); Long Pond, National Park, milieu marécageux (=marshy environment), 30-VII-1979, R. Sexton (2).

##### Diagnosis.

This species is, at present, best identified by the shape of the median lobe of the aedeagus and branches of the paramere (Fig. 499 in Smetana 1995).

**Maps 29–32.**
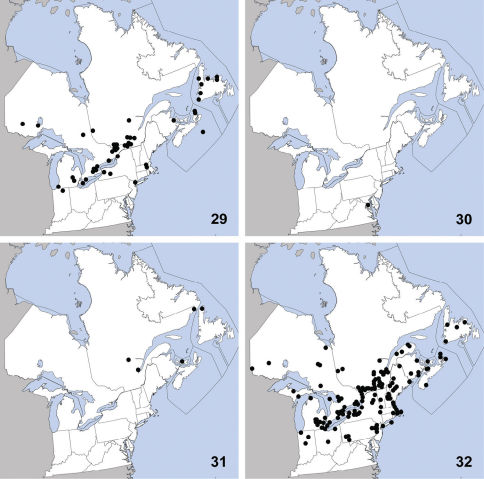
Distribution in northeastern North America, sources of data other than DEBU are quoted in parentheses. **29** Philonthus couleensis Hatch (Smetana 1995) **30** Philonthus gavius Smetana **31** Philonthus leechensis Hatch (Smetana 1995) **32** Philonthus lindrothi Smetana (Smetana 1995, Paquin and Dupérré 2001).

Philonthus couleensis was previously known from Alberta, British Columbia, Idaho, Illinois, Indiana, Manitoba, Massachusetts, Michigan, New Brunswick, Newfoundland, New Jersey, Northwest Territories, New York, Nova Scotia, Ontario, Saskatchewan, Washington, and Wisconsin (Smetana 1995). Herein we newly report it from Prince Edward Island (Map 29). This species is hygrophilous and has been collected from a variety of wet microhabitats near water (Smetana 1995).

#### Philonthus gavius

Smetana, 1995

##### Materials.

**UNITED STATES: MD:** **Prince George Co.**, Cedarville St. Pk., at lights, 24 to 29-VII-2008, S. Paiero (1).

##### Diagnosis.

Philonthus gavius is recognized among other northeastern Philonthus by the combination of: tergite eight distinctly emarginate in both sexes; elytra with most punctures separated by their widths; eyes longer than the temples.

This species was previously known from Arkansas, Illinois, Louisiana, Oklahoma, Tennessee, and Texas (Smetana 1995). It was newly recorded from Missouri by Watrous (2008) based on one specimen at a blacklight. Another specimen collected at a mercury vapour light represents a new record for Maryland (Map 30) and northeastern North America, and extends the known distribution considerably eastward. The habitat preferences of Philonthus gavius remain unknown but it is probably a species associated with wet areas similar to most other members of the Philonthus ‘Quadricollis Group’ *sensu* Smetana (1995).

#### Philonthus leechensis

Hatch, 1957

##### Materials.

**CANADA: PEI:** Brackley Beach, National Park, milieu marécageux (=marshy environment), 2-VIII-1979, R. Sexton, (3).

##### Diagnosis.

Philonthus leechensis may be recognized among other northeastern Philonthus except for Philonthus umbrinoides Smetana 1995 by the combination of: pronotum with five punctures in the dorsal row on at least one side; temples without a carina; tergite eight not emarginate in either sex; elytra without distinct markings on the disc; hind tarsus with first segment shorter than last segment; legs completely dark. From Philonthus umbrinoides it can be differentiated by the distinct, obtuse hind angles of the head.

This species was previously known from Alaska, Alberta, Arizona, British Columbia, California, Colorado, Idaho, Manitoba, Minnesota, Montana, Newfoundland, Northwest Territories, Oregon, Québec, Saskatchewan, Washington, Wisconsin, and Yukon Territory (Smetana 1995). Herein we newly record it from Prince Edward Island, representing only the fifth locality known in northeastern North America (Map 31). Philonthus leechensis appears to be a hygrophilous species, occurring mostly in northern or montane wetlands. It remains unrecorded in the eastern United States.

#### Philonthus lindrothi

Smetana, 1965

##### Materials.

**CANADA: PEI:** Brackley Beach, National Park, milieu marécageux (=marshy environment), 2-VIII-1979, R. Sexton (6).

##### Diagnosis.

Philonthus lindrothi can be distinguished fromnortheastern congeners other than Philonthus pseudolodes Smetana 1996 by the combination of: pronotum with five punctures in the dorsal row on at least one side; head with hind angles present but rounded; tergite eight not emarginate in either sex; elytra without distinct markings on the disc; hind tarsus with first segment shorter than last segment; antennae with basal segments not distinctly paler than others and with segments seven and eight elongate. Males can be readily separated from Philonthus pseudolodes by the notch in sternite eight not continuing as a grove towards its base (Smetana 1995).

This widespread species was previously known from Alaska, Alberta, Arizona, British Columbia, California, Colorado, Idaho, Illinois, Indiana, Iowa, Kansas, Maine, Manitoba, Massachusetts, Michigan, Minnesota, Missouri, Montana, Nebraska, Nevada, New Brunswick, Newfoundland, New Hampshire, New Jersey, New York, North Carolina, North Dakota, Northwest Territories, Nova Scotia, Ohio, Ontario, Oregon, Pennsylvania, Québec, Rhode Island, Saskatchewan, South Dakota, Vermont, Washington, and Wisconsin (Smetana 1995). Herein we newly record it from Prince Edward Island (Map 32). Philonthus lindrothi is an extremely common hygrophilous species that occasionally visits lights (Smetana 1995).

#### Philonthus neonatus

Smetana, 1965

##### Materials.

**UNITED STATES: VA: Giles Co.**, Ripplemead, rte 460 at bridge, flood debris, berlese, 11 to 25-V-2008, A. Brunke (4).

##### Diagnosis.

Philonthus neonatus is separated from other northeastern Philonthus by the combination of: pronotum with six punctures in both dorsal rows; pronotum no more than vaguely narrowed anteriorly; elytra distinctly red and without dark markings; elytra with micropunctures between the regular punctures; abdominal segments paler apically.

**Maps 33–36.**
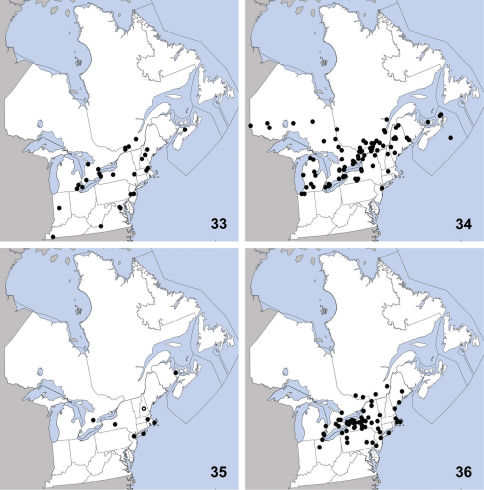
Distribution in northeastern North America, sources of data other than DEBU are quoted in parentheses. **33** Philonthus neonatus Smetana (Smetana 1995) **34** Philonthus vulgatus Casey (Smetana 1995) **35** Quedius cinctus (Paykull) (Smetana 1971, 1990, Chandler 2001, Majka et al. 2009) **36** Quedius cruentus (Olivier) (Gusarov 2001, Hoebeke 2008).

This species was previously known from Arkansas, District of Columbia, Indiana, Iowa, Kansas, Kentucky, Maine, Maryland, Massachusetts, Michigan, Mississippi, Missouri, New Hampshire, New Jersey, New York, Ontario, Pennsylvania, and Québec (Smetana 1995). Herein we newly record it from Virginia (Map 33). Philonthus neonatus is a hygrophilous species collected from debris along the margins of creeks, rivers and lakes (Smetana 1995).

#### Philonthus vulgatus

Casey, 1915

##### Materials.

**CANADA: PEI:** Brackley Beach, National Park, milieu marécageux (=marshy environment), 2-VIII-1979, R. Sexton (13).

##### Diagnosis.

This species can be easily distinguished from other northeastern Philonthus by the combination of: pronotum with punctures widely distributed and not in rows; legs and antennae completely dark; head with hind angles indistinct (Fig. 11).

Philonthus vulgatus was previously known from Alaska, Alberta, British Columbia, Idaho, Illinois, Indiana, Iowa, Kansas, Maine, Manitoba, Massachusetts, Michigan, Minnesota, Montana, Nebraska, New Hampshire, New York, North Dakota, Nova Scotia, Ontario, Québec, Saskatchewan, South Dakota, Utah, Washington, and Wisconsin (Smetana 1995). Herein we newly report this species from Prince Edward Island (Map 34). Philonthus vulgatus is a hygrophilous species that has been collected in beaver lodges, at lights, and along water margins in debris or emergent vegetation (Smetana 1995).

#### Quedius cinctus

(Paykull, 1790)

##### Materials.

**CANADA: ON: Wellington Co.**, Guelph, University Arboretum, rotting Polyporus squamosus, 29-VI-2008, A. Brunke (2); Guelph, University campus nr. horse pen, grass sweep, 13-VII-2008, C. Ho (1).

##### Diagnosis.

This species may be separated from other northeastern Quedius by the combination of: elytra with three rows of coarse punctures on the disc; head without a pair of punctures between the ocular punctures; pronotum with three punctures in each dorsal row (Fig. 12).

This Palaearctic species was first detected in North America by Smetana (1971) based on specimens collected in Massachusetts. It was subsequently recorded from New Jersey, New York and Washington by Smetana (1990), and New Hampshire by Chandler (2001). Ithas been present in North America since at least 1942 based on a collection in New Jersey and was introduced to the west coast (Washington) as early as 1979 (Smetana 1990). Majka et al. (2009) newly recorded it from Canada (New Brunswick) based on specimens collected on carrion. We here newly report Quedius cinctus from Ontario (Map 35). In its native range, Quedius cinctus is widespread in the Palaearctic region (Smetana in Löbl and Smetana 2004). This species frequents disturbed habitats throughout its range and is typically attracted to decaying organic matter; the Ontario specimens were found in a Polyporus fungus in an almost liquid state of decay.

#### Quedius cruentus

(Olivier, 1795)

##### Materials.

**CANADA: ON: Essex Co.**, Windsor, ~1.5km S Ojibway Prairie, forest-prairie edge, malaise trap, 15-V to 1-VI-2001, S. Paiero (1); Windsor, ~1.5 km S Ojibway Prairie, private prairie, malaise, 5 to 12-VI-2001, S. Paiero (3); Windsor, ~1.5km S Ojibway Prairie, private prairie, malaise, 19 to 30-VI-2001, P. Pratt (2); **Hald. –Norfolk Reg.**, Charlotte 2 Rd., ~480m E of Charlotteville, West Quarterline Rd., ‘C.C.S.N. -5’, purple prism trap, 13 to 19-VI-2009, S.M. Paiero (1); **Halton Reg.**, Milton, Derry Rd. and 4_th_ Line, under composter, 16-X-2008, S. M. Paiero (1); **Oxford Co.**, Woodstock, trails nr. river, 14-VI-2008, S.A. Marshall (1). **Simcoe Co.**, Midhurst, forest nr. Neretva St., 28-IX-2008, A. Brunke and K. Brunke (1); **Wellington Co.**, Guelph, University Campus, dairy bush, dry Polyporus squamosus, 22-IX-2008, A. Brunke (1).

##### Diagnosis.

Quedius cruentus may be distinguished from other northeastern Quedius by the combination of: elytra evenly punctate; labrum distinctly bilobed; eyes distinctly shorter than temples; antennomeres one to three distinctly paler than others; distal antennomeres strongly transverse; pronotum with sublateral row of punctures longer than dorsal row.

This Palaearctic species was first detected by Gusarov (2001) based on a specimen collected in New York. Hoebeke (2008) newly reported Quedius cruentus from Maine, Massachusetts, New Jersey, Ohio, Pennsylvania, and Québec, and established its presence in North America as early as 1983 in New York. Herein we newly report this species from Ontario (Map 36). In its native range, Quedius cruentus is widely distributed in the Palaearctic region (Smetana in Löbl and Smetana 2004). Quedius cruentus has been found in a variety of habitats in Ontario including forests, prairies, urban greenspace under loose bark, under objects and in decayed fungi.

#### Quedius curtipennis

Bernhauer, 1908

##### Materials.

**UNITED STATES: VT: Bennington Co.**, Woodford, sifting leaf litter near stream, 1-IV-2010, T. Murray (1).

**CANADA: ON:** ‘Ont.’, 30-IX-1982, G. Abayo (1); **Halton Reg.**, Oakville, nr hwy 25 and Burhamthorpe Rd., meadow, yellow pans, 12 to 14-IX-2003, S.M. Paiero (1); **Hamilton Reg.**, Hamilton, 3-VIII-1984, M.T. Kasserra (1); **Waterloo Reg.**, Blair, Fountain St. S. nr Speed River, 43.391, -80.373, hedgerow, pitfall, 28-IX-2009, A. Brunke (1); **Wellington Co.**, Eramosa, Wellington County Rds. 124 and 29, 43.615, -80.215, hedgerow, pitfall, 4-V-2010 (1), 18-V-2010 (1), 2-XI-2010 (1), A. Brunke; Guelph, 17-VIII-1976, David Levin (1); Guelph, 7-VI-1983, C.F. Langlois (1); Guelph, University Arboretum, hand collected, 16-III-1983, L.B. Carlson (1); Guelph, 30-IX-1983, A. Harris (1); Guelph, 5-VII-1984, ‘maple’, T. Young (1); Guelph, 3-IV-1991, M. Kovacevick (1); Guelph, 14-X-1998, T. Phillips (1); Guelph, Victoria Rd. and Conservation Line, 43.580, -80.275, hedgerow, pitfall, 19-V-2009 (1), 17-XI-2009 (1), A. Brunke.

**Figure 13.**
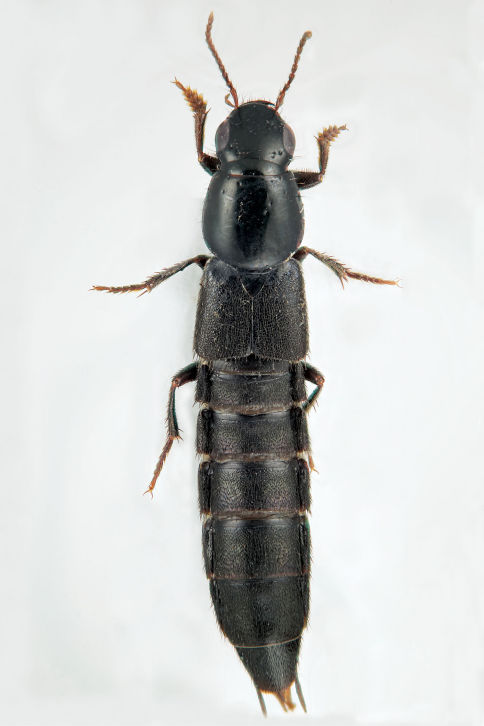
Dorsal habitus of Quedius curtipennis Bernhauer.

**Diagnosis.** Quedius curtipennis can be distinguished from other northeastern Quedius by the combination of: elytra with even punctation; labrum not bilobed; scutellum impunctate; basal antennomeres not distinctly darker than the other segments (Fig. 13).

**Maps 37–38.**
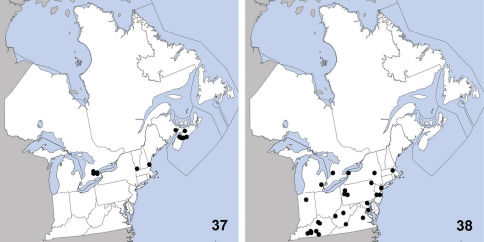
Distribution in northeastern North America, sources of data other than DEBU are quoted in parentheses. **37** Quedius curtipennis Bernhauer (Smetana 1990, Majka and Smetana 2007, Majka and Klimaszewski 2008b) **38** Quedius fulgidus (Fabricius) (Smetana 1971, Peck and Thayer 2003).

**Figures 14–19.**
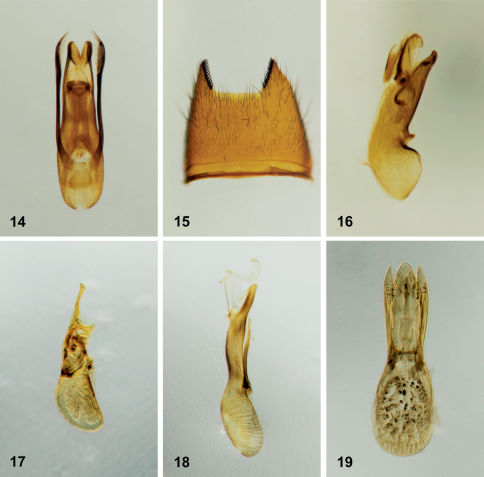
**14** Stenus clavicornis (Scopoli), aedeagus in parameral view **15** Medon fusculus (Mannerheim), 7_th_ sternite **16** Medon fusculus, aedeagus in lateral view **17** Scopaeus minutus Erichson, aedeagus in lateral view **18** Erichsonius nanus (Horn), aedeagus in lateral view **19** Erichsonius parcus (Horn), aedeagus in parameral view.

This exotic Palaearctic species was first correctly reported in North America by Korge (1962) who recognized that Quedius parallelus Hatch 1957, a species described from western North America, was synonymous with Quedius curtipennis Bernhauer 1908. Quedius curtipennis has been present in North America as early as 1934 based on specimens from Washington and also occurs in British Columbia and Oregon (Smetana 1971). A separate introduction to the east was detected first by Smetana (1990) based on a New Hampshire specimen collected in 1983. Since then Quedius curtipennis has been detected in New Brunswick and Nova Scotia (Majka and Smetana 2007). Herein we newly report it from Ontario and Vermont (Map 37) from specimens collected as early as 1976 and 2010, respectively. The record from Guelph, Ontario represents the earliest known collection in eastern North America. In its native range, Quedius curtipennis is widely distributed in the Palaearctic region (Smetana in Löbl and Smetana 2004). Quedius curtipennis has been collected in Ontario mainly in disturbed habitat such as regenerating woodland, fields and agricultural crops.

#### Quedius fulgidus

(Fabricius, 1793)

##### Materials.

**CANADA: ON: Huron Co.**, Seaforth, 7-VII-1955, D. Keys (1).

##### Diagnosis.

Quedius fulgidus may be distinguished from other northeastern Quedius by the combination of: elytra evenly punctate; labrum distinctly bilobed; eyes distinctly shorter than temples; antennomeres one to three not distinctly paler than others; distal antennomeres only slightly transverse; pronotum with sublateral row of punctures longer than dorsal row.

This Palaearctic species was first correctly recognized in North America by Horn (1878) who synonymised Quedius iracundus (Say 1834), a species described from North America, with Quedius fulgidus; this synonymy was later confirmed by Smetana (1971). The first verifiable specimens from North America were collected in 1874 from Iowa (Smetana) but as Quedius iracundus (*=Q. fulgidus*)was described in 1834 from Indiana, Quedius fulgidus has surely been present long before 1874. Currently, Quedius fulgidus is known from Arizona, British Columbia, California, Colorado, District of Columbia, Georgia, Idaho, Illinois, Indiana, Kansas, Kentucky, Manitoba, Maryland, Massachusetts, Michigan, Minnesota, Mississippi, Missouri, New Jersey, New Mexico, New York, Oregon, Pennsylvania, Texas, Virginia, Washington, West Virginia, Wisconsin (Smetana 1971), and Tennessee (Smetana 1978). Herein we newly record this species from eastern Canada (Ontario) (Map 38). This species is strongly synanthropic in North America (Smetana 1971) but occurs regularly in caves in the more southern portion of its range (Peck and Thayer 2003). In its native range Quedius fulgidus is widely distributed in the Palaearctic region (Smetana in Löbl and Smetana 2004). Quedius fulgidus appears to be uncommon in the northeastern extreme of its range compared to its close relative Quedius cruentus, another exotic species that was recently established on the continent (see above) and has since become extremely common.

## General discussion

Curation of over 32,000 staphylinids deposited in the University of Guelph Insect Collection resulted in the discovery of thirty-five new provincial or state records, six new Canadian records, one new record for the United States and two new records for eastern Canada. Many of these specimens were aleocharines and a future publication is planned to report on the discoveries made while curating this subfamily. The majority of the records presented herein involved species of which were included in recent revisions (after 1970), suggesting that even of ‘well-known’ groups, our knowledge of staphylinid distributions remains incomplete. Two boreal beetles, Porrhodites fenestralis and Bisnius cephalicus were newly recorded in Ontario, joining previous records from adjacent provinces to suggest a transcontinental distribution. Six staphylinid species were newly discovered in Canada at the northern extreme of their range, two of which are apparently entirely restricted in Canada to the Carolinian ecoregion (Sepedophilus versicolor and Erichsonius parcus). This relatively small area of southernmost Ontario is the most biodiverse region in Canada (Ontario Ministry of Natural Resources 2010) and has yielded a multitude of new records for Canada in other insect groups (e.g., Orthoptera: Marshall et al. 2004; Hemiptera: Paiero et al. 2003; and aculeate Hymenoptera: Buck 2003, Buck et al. 2005); however it is heavily impacted by agriculture and development (Ontario Ministry of Natural Resources 2010). The distributions of four rare or infrequently collected species (Sepedophilus campbelli, Tachyporus browni, Eustilicus tristis, and Bisnius cephalicus) in northeastern North America were previously fragmentary and were augmented by new data presented herein. Ten exotic species for which we give new state, or provincial, or national records are apparenly appear to be expanding their range in northeastern North America, and have the potential to become widespread across the continent. One of these species, Tachinus corticinus, was first collected in North America in 1967 but now dominates autumn leaf litter staphylinid assemblages in Ontario woodland fragments where it can comprise up to 47% of all individuals (A. Brunke, *unpublished data*). Levesque and Levesque (1995) found that this species made up as much as 18% of all staphylinid individuals in Québec raspberry plantations. The impact of this abundance on native North American biodiversity is unknown but may be substantial. New collection data for Quedius curtipennis establishes its presence in eastern North America as early as 1976, seven years earlier than previously known.

The results of this paper demonstrate the key role of curated insect collections in understanding biodiversity in the boreal region, the imperilled ‘Carolinian’ region in Canada, and northeastern North America in general. An improved understanding of rare or or potentially rare insect species, and the effective detection of exotic species, depends on the routine identification of specimens in collections and the regular implentation of regional insect surveys. We recommend increased support for these activities to develop and maintain a clear picture of biodiversity, and biodiversity change, in northeastern North America.

## Supplementary Material

XML Treatment for Omalium repandum

XML Treatment for Porrhodites fenestralis

XML Treatment for Ischnosoma flavicolle

XML Treatment for Sepedophilus campbelli

XML Treatment for Sepedophilus marshami

XML Treatment for Sepedophilus occultus

XML Treatment for Sepedophilus opicus

XML Treatment for Sepedophilus versicolor

XML Treatment for Tachinus corticinus

XML Treatment for Tachyporus browni

XML Treatment for Tachyporus ornatus

XML Treatment for Bledius neglectus

XML Treatment for Stenus clavicornis

XML Treatment for Eustilicus tristis

XML Treatment for Medon fusculus

XML Treatment for Scopaeus minutus

XML Treatment for Sunius melanocephalus

XML Treatment for Acylophorus agilis

XML Treatment for Bisnius cephalicus

XML Treatment for Bisnius siegwaldii

XML Treatment for Erichsonius brachycephalus

XML Treatment for Erichsonius nanus

XML Treatment for Erichsonius parcus

XML Treatment for Gabrius amulius

XML Treatment for Gabrius appendiculatus

XML Treatment for Gabrius vindex

XML Treatment for Neobisnius occidentoides

XML Treatment for Neobisnius terminalis

XML Treatment for Philonthus couleensis

XML Treatment for Philonthus gavius

XML Treatment for Philonthus leechensis

XML Treatment for Philonthus lindrothi

XML Treatment for Philonthus neonatus

XML Treatment for Philonthus vulgatus

XML Treatment for Quedius cinctus

XML Treatment for Quedius cruentus

XML Treatment for Quedius curtipennis

XML Treatment for Quedius fulgidus
